# Impacts of leaf age and heat stress duration on photosynthetic gas exchange and foliar nonstructural carbohydrates in *Coffea arabica*

**DOI:** 10.1002/ece3.2681

**Published:** 2017-01-29

**Authors:** Danielle E. Marias, Frederick C. Meinzer, Christopher Still

**Affiliations:** ^1^ Department of Forest Ecosystems and SocietyOregon State UniversityCorvallisORUSA; ^2^ USDA Forest ServicePacific Northwest Research StationCorvallisORUSA

**Keywords:** chlorophyll fluorescence, heat stress, nonstructural carbohydrates, photosynthesis, stomatal conductance

## Abstract

Given future climate predictions of increased temperature, and frequency and intensity of heat waves in the tropics, suitable habitat to grow ecologically, economically, and socially valuable *Coffea arabica* is severely threatened. We investigated how leaf age and heat stress duration impact recovery from heat stress in *C. arabica*. Treated plants were heated in a growth chamber at 49°C for 45 or 90 min. Physiological recovery was monitored in situ using gas exchange, chlorophyll fluorescence (the ratio of variable to maximum fluorescence, *F*_V_/*F*_M_), and leaf nonstructural carbohydrate (NSC) on mature and expanding leaves before and 2, 15, 25, and 50 days after treatment. Regardless of leaf age, the 90‐min treatment resulted in greater *F*_V_/*F*_M_ reduction 2 days after treatment and slower recovery than the 45‐min treatment. In both treatments, photosynthesis of expanding leaves recovered more slowly than in mature leaves. Stomatal conductance (*g*_s_) decreased in expanding leaves but did not change in mature leaves. These responses led to reduced intrinsic water‐use efficiency with increasing heat stress duration in both age classes. Based on a leaf energy balance model, aftereffects of heat stress would be exacerbated by increases in leaf temperature at low *g*_s_ under full sunlight where *C. arabica* is often grown, but also under partial sunlight. Starch and total NSC content of the 45‐min group significantly decreased 2 days after treatment and then accumulated 15 and 25 days after treatment coinciding with recovery of photosynthesis and *F*_V_/*F*_M_. In contrast, sucrose of the 90‐min group accumulated at day 2 suggesting that phloem transport was inhibited. Both treatment group responses contrasted with control plant total NSC and starch, which declined with time associated with subsequent flower and fruit production. No treated plants produced flowers or fruits, suggesting that short duration heat stress can lead to crop failure.

## 1 Introduction

Climate models predict an increasing frequency and intensity of heat waves and high temperature events throughout the 21st century (Cramer et al., 2001; Diffenbaugh & Scherer, 2011; IPCC, 2014) that are expected to influence plant species' distributions, productivity, and carbon balance, although the physiological impacts remain unclear. Heat waves are increasing under both drier and wetter conditions (Hao, AghaKouchak, & Phillips, 2013). In contrast to drought, much less is known about physiological responses to heat stress (Barnabás, Jäger, & Fehér, 2008; Ruan, Jin, Yang, Li, & Boyer, 2010). This, along with the need to isolate the effects of heat and drought stress to understand the interactions between the two on plant physiological responses (Sevanto & Dickman, 2015), makes studies on the physiological impacts of heat stress essential. Tropical species are particularly vulnerable to heat stress because of the higher radiation load, the increase in heat wave intensity and frequency expected in the tropics (Corlett, 2011), and the narrower distribution of temperatures typically experienced compared to extratropical species (Battisti & Naylor, 2009).

The tropics support important agricultural crops such as coffee. Global coffee consumption continues to increase; over 9 billion kg of coffee was consumed worldwide in 2014 (ICO, 2016). *Coffea arabica* L. leads the world coffee trade and provides ~65% of commercial production (ICO, 2016). *Coffea arabica* is a shade‐adapted evergreen species that originated in Ethiopia and is now grown in 80 countries on four continents in the intertropical zone between 20 and 25°N in Hawaii and 24°S in Brazil (DaMatta & Ramalho, 2006). *Coffea arabica* is highly sensitive to fluctuations in temperature (Camargo, 2010; Silva, DaMatta, Ducatti, Regazzi, & Barros, 2004) with an optimal mean annual temperature range of 18–24°C (Camargo, 1985; Teketay, 1999). An increase in the frequency and intensity of heat waves in the tropics would severely threaten suitable habitat to grow *C. arabica* (Bunn, Läderach, Rivera, & Kirschke, 2014; de Camargo, 2010; Craparo, Van Asten, Läderach, Jassogne, & Grab, 2015; DaMatta & Ramalho, 2006; Davis, Gole, Baena, & Moat, 2012). Therefore, it is crucial to quantify its physiological responses to and ability to recover from heat stress (Martins, Tomaz, Lidon, DaMatta, & Ramalho, 2014; Martins et al., 2016; Rodrigues et al., 2016).

Examining the impacts of heat stress on plant carbon utilization is critical for understanding plant responses to changes in climate and potential feedbacks between vegetation and climate. Heat stress affects plant physiology from the cellular to whole plant scales, inducing shifts in the allocation of assimilated carbon/photosynthate to repair and recovery processes. At the cellular and organelle levels, high temperatures can damage photosystem II (PSII) photochemistry and electron transport; reduce thylakoid membrane fluidity, RUBISCO activity, and cell membrane stability; and induce heat‐shock protein expression and the production of reactive oxygen species (ROS) (Teskey et al., 2015; Wahid, Gelani, Ashraf, & Foolad, 2007). At the leaf level, high temperature stress reduces photosynthesis, increases photorespiration and dark (mitochondrial) respiration, and influences water relations and stomatal conductance (Wahid et al., 2007). At the whole plant level, heat stress impacts leaf area, leaf abscission, visible foliar damage (Cunningham & Read, 2006), budburst, growth, mortality, and reproduction (Teskey et al., 2015). In response to high temperature stress, plants use assimilated carbon/photosynthate to produce compounds used for repair, defense, and physiological recovery such as primary and secondary metabolites, antioxidants, osmolytes, and phytohormones (Bita & Gerats, 2013). However, we have limited knowledge of how plant carbon allocation is altered in response to heat stress.

Nonstructural carbohydrates (NSCs) traditionally include starch and free sugars (sucrose, glucose, and fructose) and are involved in growth, storage, reproduction, metabolism, and repair (Kozlowski, 1992). Although NSCs play a role in plant responses to environmental stress (Dietze et al., 2014) and it is understood that high temperature shifts carbon metabolism enzymes, starch accumulation, and sucrose synthesis (Bita & Gerats, 2013; Ruan et al., 2010), the role of NSCs in the response to and recovery from heat stress is poorly understood due to conflicting results (Génard et al., 2008; Sala, Woodruff, & Meinzer, 2012). For example, NSCs may increase in response to heat stress because NSCs are used for repair and damage prevention (Couée, Sulmon, Gouesbet, & Amrani, 2006; Roitsch & González, 2004; Sevanto & Dickman, 2015; Sugio, Dreos, Aparicio, & Maule, 2009) and therefore are associated with heat stress tolerance (Liu & Huang, 2000; Niinemets, 2010). In contrast, leaf NSCs have also been shown to decrease in response to heat stress due to reduced carbon gain and assimilation (i.e., decreased supply) by inhibited photosynthesis and stomatal conductance (Zhao, Hartmann, Trumbore, Ziegler, & Zhang, 2013) and/or increased utilization (i.e., increased demand) by increased respiration and metabolic maintenance (Duan et al., 2013).

These divergent observations of NSC responses to heat stress may be due to variation in the severity of the heat stress, which influences the extent of damage and the capacity to recover. Based on the findings of Bauweraerts et al. (2013, 2014), episodic heat wave events produced more stress than a constant increase in temperature, emphasizing the importance of considering duration and intensity of heat stress when predicting plant responses and the capacity to recover. Heat stress severity is a function of intensity (exposure temperature) and duration of exposure (Colombo & Timmer, 1992). Bauweraerts et al. (2014) found that growth of *Quercus* seedlings increased in a + 6°C treatment but decreased in a + 12°C treatment. Therefore, it is expected that NSC dynamics would also be influenced by heat stress severity, although this remains unknown. Given that even short heat events can have substantial impacts on carbon gain (Filewod & Thomas, 2014), the predicted fluctuations in the duration of summer heat waves (Della‐Marta, Haylock, Luterbacher, & Wanner, 2007), and the paucity of studies manipulating heat stress duration, research on the effects of heat stress duration on physiological recovery and NSC dynamics is needed. NSC dynamics in *C. arabica* are tightly linked to sink demand from vegetative and reproductive growth (Chaves, Martins, Batista, Celin, & DaMatta, 2012; Génard et al., 2008; Ramalho et al., 2013), and many studies have investigated whether NSC levels regulate *C. arabica* photosynthesis (Batista et al., 2011; DaMatta et al., 2016; Franck, Vaast, Génard, & Dauzat, 2006; Ronchi et al., 2006; Vaast, Angrand, Franck, Dauzat, & Génard, 2005). However, to our knowledge, no study has investigated the impacts of heat stress‐induced reductions in photosynthesis as a cause rather than a consequence of NSC dynamics in *C. arabica*.

Previous work has shown that thermotolerance of tropical species measured with chlorophyll fluorescence increases with leaf age, an evolutionary adaptation to protect older and longer‐lived foliage from irreversible damage (Yamada, Hidaka, & Fukamachi, 1996; Zhang, Poorter, Hao, & Cao, 2012). This pattern was also observed on detached *C. arabica* leaf disks (Marias, Meinzer, & Still, 2016). Because NSCs have been linked to heat stress responses and heat tolerance (Liu & Huang, 2000), NSCs may influence the ability of plants to avoid permanent damage, to tolerate heat stress, and/or to recover from heat stress. Therefore, leaf age‐related differences in thermotolerance and the ability to recover from heat stress may be related to NSC dynamics (Filewod & Thomas, 2014; Teskey et al., 2015). However, this has not been investigated in *C. arabica* plants in situ.

The goal of this study was to investigate how leaf age and heat stress duration influence NSC dynamics and physiological responses to and recovery from heat stress in *C. arabica*. We exposed treated plants to a simulated sudden heat wave at 49°C in a growth chamber for two different heat stress durations (45, 90 min) and monitored physiological responses and recovery in expanding and mature leaves for 50 days after treatment using gas exchange, chlorophyll fluorescence, and leaf NSCs. Because *C. arabica* is grown in both sun and shade (DaMatta, Ronchi, Maestri, & Barros, 2007) and to put this study in the context of a combined heat and drought scenario, we used a leaf energy balance model to investigate the effects of reduced stomatal conductance on leaf temperature in partial and full sun conditions. We hypothesized that 1) mature leaves would exhibit less physiological damage and/or faster recovery than expanding leaves, and 2) the 90‐min heat stress duration would result in greater physiological damage and/or slower recovery than the 45‐min heat stress duration.

## 2 Materials and Methods

### 2.1 Plant material

*Coffea arabica* L. (Eritrean Mokka) plants about 6–9 months old, obtained from the Hawaii Agriculture Research Center in January 2014, were grown in a peat–perlite–pumice growing mix (Sunshine LA4P) in 9.6‐L pots in a glasshouse in Corvallis, Oregon. Supplemental metal halide and high‐pressure sodium lighting (400 watts) was used to maintain a 12‐hour photoperiod during fall and winter months. The first round of experiments began 21 July 2014, and the second round began 19 August 2014. Plants were ~1 m tall. During the sampling rounds, average daytime temperature was 23.5°C, average nighttime temperature 18.1°C, average daytime relative humidity 64%, average nighttime relative humidity 79%, and average daily maximum photosynthetically active radiation (PAR) 325 μmol·m^−2^·s^−1^. Due to controlled glasshouse conditions, temperature, relative humidity, and PAR did not substantially differ between rounds (Table S1, Figure S1). Plants were kept well‐watered and fertilized once every 2 weeks (Miracle‐Gro All‐Purpose Liquid Plant Food, 12% N, 4% P_2_O_5_, 8% K_2_O).

*Coffea arabica* is a tropical evergreen species that continually produces new flushes of leaves year‐round. Leaf age class (expanding, mature) was determined by visually dividing a mid‐canopy plagiotropic branch into thirds where leaf age sequence increased from the outermost leaves to the base of the branch (e.g., Wright, Leishman, Read, & Westoby, 2006). Leaves in the outer third were the youngest and still expanding (expanding), and leaves in the inner third were the oldest, fully expanded, and mature (mature). Mean photosynthesis values were 6.3 and 7.0 μmol·m^−2^·s^−1^ for expanding and mature leaves, respectively, consistent with previously reported values for *C. arabica* (DaMatta et al., 2007).

### 2.2 Heat stress duration treatments

Plants were exposed to 49°C in a growth chamber (Model I‐35LVL, Percival, Boone, IA) that accommodated two plants at a time and was equipped with cool white lighting (PAR = 25 μmol·m^−2^·s^−1^). The treatment temperature of 49°C was selected based on the temperature at which a 50% reduction in initial chlorophyll fluorescence occurred (49.0 ± 0.5°C) on *C. arabica* leaf disks (Marias et al., 2016). Preliminary experiments at other temperatures also showed that 49°C induced enough heat stress to be damaging without completely scorching/killing leaves, allowing us to monitor recovery. Plants were watered to drainage directly before treatment to avoid drought effects and to buffer changes in soil temperature during treatment. Fine‐wire thermocouples measured air, leaf, and soil (~10 cm depth) temperatures during treatment exposure (Figure S2). Pots were completely wrapped with reflective bubble wrap to isolate the soil and roots from heat exposure. This prevented soil temperatures from exceeding 30°C (Figure S2), which is realistic for soil temperatures in summer (Zheng, Hunt, & Running, 1993). Two plants were heated for 45 min, and two different plants were heated for 90 min on the same day in two experimental rounds: one on 21 July 2014 and one on 19 August 2014 (*N* = 2 per round). Control (0 min) plants were not exposed to a temperature treatment (*N* = 4 per round). Rounds were combined (*N* = 4 for treated plants, *N* = 8 for controls) because environmental conditions (Table S1) and physiological measurements did not differ between rounds. The growth chamber did not have the capability to adjust light levels. Although light can influence chlorophyll fluorescence (Buchner, Karadar, Bauer, & Neuner, 2013; Ludlow, 1987), we assumed that the low light levels for the 45‐min or 90‐min treatment duration did not substantially influence results. Photosynthesis, chlorophyll fluorescence, and foliar NSC content were monitored in control and treated plants prior to treatment (day 0) and 2, 15, 25, and 50 days after treatment. Three leaves per leaf age class per individual plant were marked for resampling. When leaf drop occurred, an intact leaf was selected and marked for resampling for the remainder of the experiment.

### 2.3 Photosynthesis and stomatal conductance measurements

Photosynthesis and stomatal conductance were measured in the morning during active gas exchange (before afternoon stomatal closure occurred) between 0700 and 1,000 hr (dawn was ~0500 hr) on 1–3 marked leaves per leaf age class per individual plant using a portable photosynthesis system (LI‐6400, Li‐Cor, Lincoln, NE, USA). The ratio of photosynthesis to stomatal conductance (*A*/*g*_*s*_), an estimate of intrinsic water‐use efficiency (i*WUE*, Jones, 2009), was calculated. In the cuvette, PAR was set to 500 μmol·m^−2^·s^−1^, leaf temperature was set to 25°C, [CO_2_] sample was set to 400 μmol/mol (to represent ambient atmospheric [CO_2_]), and flow rate was set to 500 μmol/s. Day 0 photosynthesis values were estimated from photosynthesis‐intercellular CO_2_ (*C*_i_) curves. To compare intrinsic photosynthesis at the ambient atmospheric [CO_2_] of 400 μmol/mol, photosynthesis was estimated at the average *C*_i_ value of the control (0 min) group averaged over all sampling days, which was 246 μmol/mol for expanding leaves and 254 μmol/mol for mature leaves.

### 2.4 Chlorophyll fluorescence measurements

Chlorophyll fluorescence was measured on 1–3 marked leaves per leaf age class per individual plant at ambient temperature with a portable pulse–amplitude modulated chlorophyll fluorometer (Mini‐PAM, Heinz Walz Gmbh, Germany) at predawn to ensure leaves were dark‐adapted. Chlorophyll fluorescence was measured as the ratio of variable to maximum fluorescence (*F*_V_/*F*_M_) in the convention of Maxwell and Johnson (2000). *F*_V_/*F*_M_ measures the maximum quantum efficiency of PSII photochemistry (Genty, Briantais, & Baker, 1989) and is calculated as:

(1) $$\frac{F_{V}}{F_{M}}=\frac{F_{M}-F_{O}}{F_{M}}=1-\frac{F_{O}}{F_{M}}$$

A measuring light (red light‐emitting diode, 650 nm, 0.15 μmol·m^−2^·s^−1^ PAR) with a pulse–width of 3 μs was turned on at a pulse modulation frequency of 0.6 kHz to induce the minimal level of fluorescence (*F*_O_). *F*_V_/*F*_M_ was then determined by applying a 0.8 s saturating pulse of white light (18,000 μmol photons·m^−2^·s^−1^ PAR), which transiently closed all PSII reaction centers (preventing any photochemical processes from occurring), minimized heat dissipation (since leaves were dark‐adapted), and induced maximum and variable fluorescence.

### 2.5 Nonstructural carbohydrate (NSC) analysis

One leaf per age class per individual plant was collected early morning (directly after *F*_V_/*F*_M_ measurements were made) on each sampling date, immediately put on ice in a cooler, and transported to the nearby laboratory where samples were microwaved for 90 s to stop all enzymatic activity and oven‐dried at 75°C. Samples were stored in a freezer before being ground to a fine powder. Leaf samples were analyzed for content of total NSC, starch, sucrose, and glucose + fructose as described by Woodruff and Meinzer (2011). Water was added to the powdered samples, and NSC was extracted from the solutions by heating them in steam for 1.5 hr. The concentration of free glucose + fructose was determined photometrically on a 96‐well microplate photometer (Multiskan FC, Thermo Scientific, Waltham, MA, USA) after enzymatic conversion of glucose + fructose to gluconate‐6‐phosphate. Samples were hydrolyzed by enzymatic treatment: invertase for sucrose and amyloglucosidase for total NSC. Photometric analysis was based on absorbance of samples at 340 nm in solution with reference to the absorbance of a glucose reference solution. Total NSC was calculated as the sum of starch, sucrose, and glucose + fructose. NSC values (mg/g dry weight) are presented in figures as % dry weight.

### 2.6 Leaf energy balance model

A simple leaf energy balance model created by Kevin Tu (http://landflux.org/Tools.php) was used to estimate the effect of shifts in stomatal conductance (*g*_s_) on leaf temperature (*T*_leaf_). The modified leaf energy balance equation from Sridhar and Elliott (2002), Monteith and Unsworth (2007), and Jones (2013) is described in detail in Supporting Information and Table S2. To investigate the effect of changes in *g*_s_ at representative heat wave temperatures potentially experienced by *C. arabica* in the tropics, air temperatures (*T*_air_) of 35°C, 40°C, and 45°C were used in the model. The range of *g*_s_ values used was 0–0.15 mol·m^−2^·s^−1^, similar to that observed in this study as well as previously reported values for field grown *C. arabica* (Meinzer et al., 1990; Gutierrez & Meinzer, 1994; Silva et al., 2004). Environmental and leaf parameters were set to represent conditions experienced by *C. arabica* in the tropics: Short‐wave radiation (SWR) was 600 and 1,000 W/m^2^ to simulate partial and full sun conditions, respectively, wind speed was 2.0 m/s, relative humidity was 65%, leaf angle was 20° from horizontal, absorptance to SWR was 0.50, emissivity was 0.96, and leaf length in the direction of wind was 100 mm. Incident photosynthetically active radiation (PAR) for each SWR level at a wavelength of 550 nm (average wavelength for the 400–700 nm PAR range) was ~1,380 and ~2,300 μmol·m^−2^·s^−1^, respectively. PAR is assumed ~50% of SWR (Britton & Dodd, 1976).

### 2.7 Statistical analysis

A three‐way factorial linear mixed‐effects model was developed with leaf age, treatment, and day as main fixed effects. Nested random effects in the model were plant and leaf within plant. Response variables were photosynthesis, stomatal conductance, i*WUE*,* F*_V_/*F*_M_, total NSC, starch, sucrose, and glucose + fructose. To choose a correlation structure that would account for the repeated measurements of leaves within plants through time, four models that allowed for different residual correlation structures were fit and selected based on the minimum Bayesian information criterion (BIC) value. Assumptions of constant variance and normality were checked using residual and quantile–quantile plots. Log‐transformations were necessary to meet assumptions for starch and glucose + fructose. For ease of interpretation, we present back‐transformed data in results and figures. All interactive and main effects of factors on the response were tested using marginal F‐tests (also known as type III tests) that account for unbalanced sample sizes. Post hoc comparisons were made using a 95% confidence interval and *p* < .05. Due to sufficient degrees of freedom, we did not make multiple comparisons corrections. If no significant differences between leaf age classes existed, NSC components of expanding and mature leaves were combined by averaging over leaf age to simplify data visualization.

The slope of *F*_V_/*F*_M_ recovery was determined for each plant by fitting a line of best fit (linear regression) from the *F*_V_/*F*_M_ value 2 days after treatment (i.e., the minimum *F*_V_/*F*_M_ value or maximum damage after treatment) through the day at which *F*_V_/*F*_M_ recovered to day 0 values. The slope of recovery was log‐transformed, and its relationship with the *F*_V_/*F*_M_ 2 days after treatment was described by a logarithmic nonlinear regression equation (*f* = *y*_0_ + a*ln(abs(*x*)). A two‐way factorial linear mixed‐effects model was developed with leaf age and treatment as main fixed effects for slope of recovery and minimum *F*_V_/*F*_M_ as response variables. Procedures to select a correlation structure and check assumptions were the same as stated above.

Pearson product–moment correlation was used to quantify the strength of the relationship between photosynthesis and each NSC component (total NSC, starch, sucrose, glucose + fructose) within each treatment group (control (0 min), 45, 90 min) for all days sampled. Statistical analyses were conducted in SigmaPlot 13.0 (Systat Software, San Jose, CA, USA) and R version 3.2.3 (2015‐12‐10, The R Foundation for Statistical Computing).

## 3 Results

Both the 45‐ and 90‐min treatments induced significant shifts in gas exchange, *F*_V_/*F*_M_, and NSC content and dynamics. It was also observed that after the experiment, the controls (0 min) produced flowers and fruits, whereas the 45‐ and 90‐min treatment groups did not.

### 3.1 Gas exchange and *F*_V_/*F*_M_

**Figure 1:**
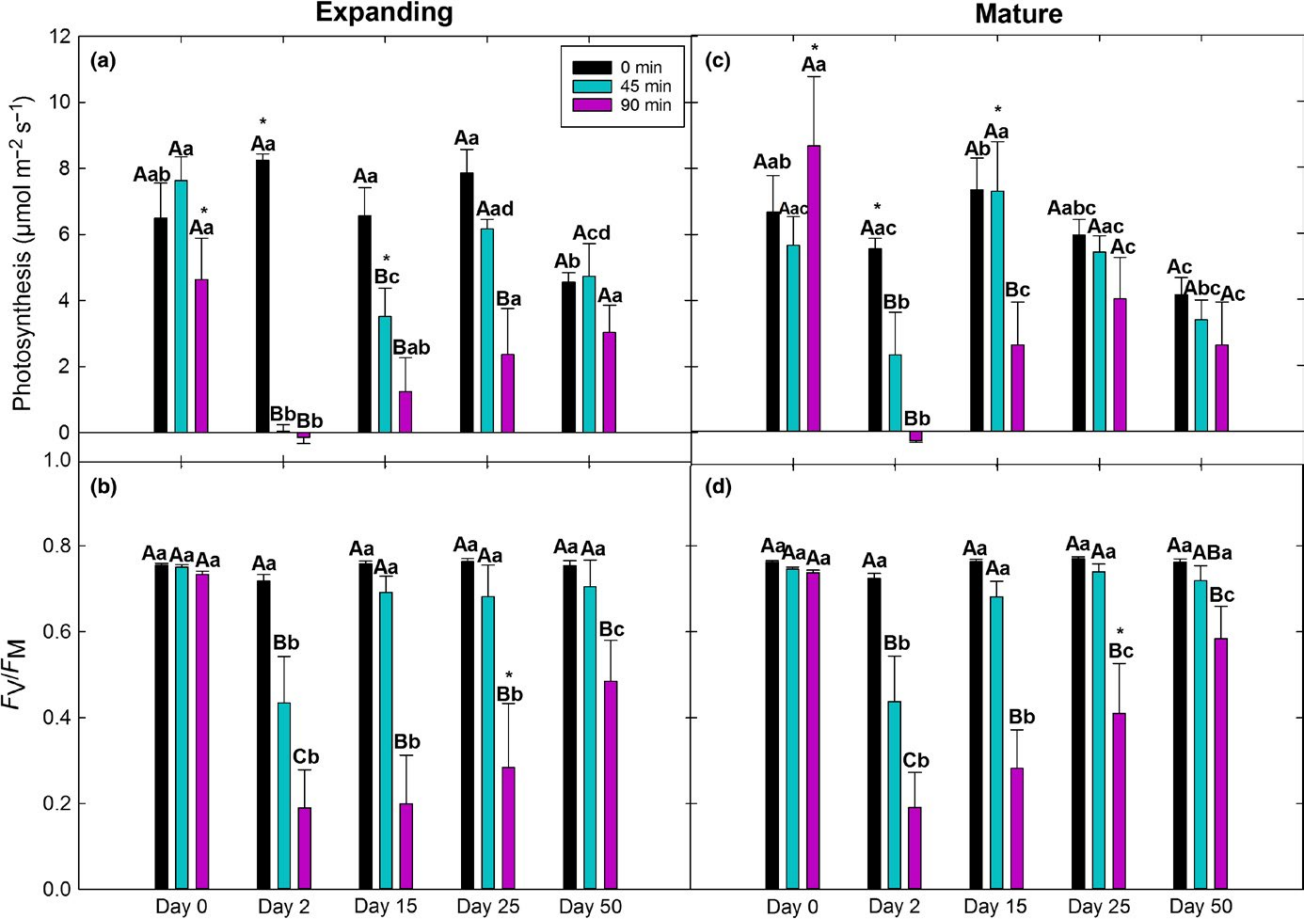
Time courses of photosynthesis and *F*_V_/*F*_M_ of expanding (a, b) and mature (c, d) leaves from the same plants (*N* = 4) of the 0‐min (controls), 45‐min, and 90‐min groups at 0, 2, 15, 25, and 50 days after treatment. Different uppercase letters represent significant differences among treatments within each day and leaf age. Different lowercase letters represent significant differences among days within treatment and leaf age. Asterisks represent significant differences between leaf age within treatment and day. Error bars represent ± *SE*

Interactions among the main effects (age, treatment, day) on photosynthesis and *F*_V_/*F*_M_ are summarized in Table 1. Photosynthesis significantly declined 2 days after treatment in both the 45‐ and 90‐min groups for both expanding and mature leaves but slower recovery to control values in the 90‐min group compared to the 45‐min group (Figure 1a,c). Photosynthesis of mature leaves in the 45‐min group recovered to control values by day 15 while that of the 90‐min group recovered by day 25. In contrast, expanding leaves took longer to recover to control values than mature leaves regardless of treatment with photosynthesis of the 45‐min group recovering by day 25 and that of the 90‐min group recovering by day 50. Within‐day and within‐treatment differences between leaf age classes were variable. Like photosynthesis, *F*_V_/*F*_M_ also declined 2 days after treatment, yet the amount of the reduction in *F*_V_/*F*_M_ 2 days after treatment was significantly greater in the 90‐min group than the 45‐min group in both leaf age classes (Figure 1b,d). Regardless of leaf age class, *F*_V_/*F*_M_ of the 45‐min group recovered to control values by day 15, while the 90‐min group did not fully recover by day 50, similar to the slower recovery of the 90‐min group compared to the 45‐min group as measured with photosynthesis.

**Table 1:** Marginal F‐tests for photosynthesis and *F*_V_/*F*_M_

	Photosynthesis (μmol·m^−2^·s^−1^)				*F*_V_/*F*_M_			
	numDF	denDF	*F*‐value	*p*‐Value	numDF	denDF	*F*‐value	*p*‐Value
Intercept	1	93	366.41	**<.0001**	1	104	883.02	**<.0001**
Age	1	13	1.6028	.2277	1	13	6.846	**.0213**
Treatment	2	13	21.357	**.0001**	2	13	26.312	**<.0001**
Day	4	93	20.077	**<.0001**	4	104	94.940	**<.0001**
Age × Treatment	2	13	3.4666	.0621	2	13	2.8695	.0929
Age × Day	4	93	3.8058	**.0065**	4	104	1.2742	.2849
Treatment × Day	8	93	8.2791	**<.0001**	8	104	38.442	**<.0001**
Age × Treatment × Day	8	93	3.5207	**.0013**	8	104	0.6461	.7373

*N* = 4. Bolded *p*‐values indicate *p* < .05. NumDF and denDF are the degrees of freedom in the numerator and denominator, respectively.

**Figure 2:**
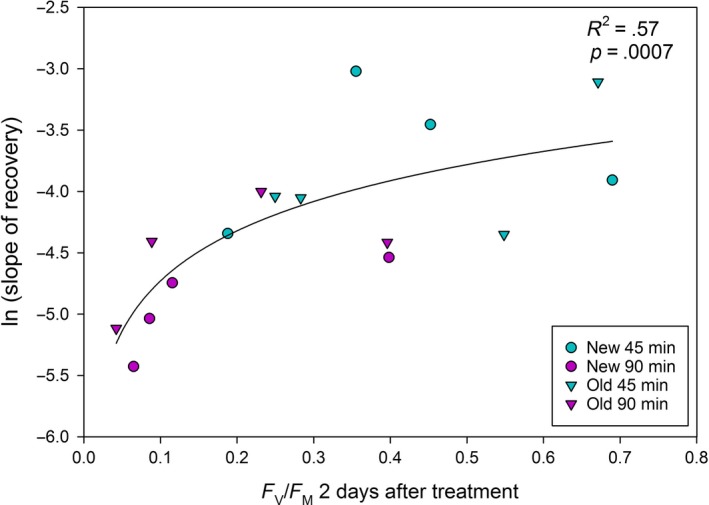
*F*_V_/*F*_M_ values reached 2 days after treatment (i.e., maximum damage after treatment) significantly related to the log‐transformed slope of *F*_V_/*F*_M_ recovery across leaf age class and treatment (*R* ^2^ = .57, *p* = .0007). Nonlinear regression equation: *f* = *y*_0_ + a*ln(abs(*x*). Each data point represents an individual plant. *N* = 4

The log‐transformed slope of recovery (ln(slope of recovery) back to control values significantly decreased with increasing damage assessed with *F*_V_/*F*_M_ 2 days after treatment (*p* = .0007, *R* ^2^ = .57, Figure 2). Mean ln(slope of recovery) did not differ among leaf age classes (*p* = .86) but significantly differed between treatments where the 90‐min treatment exhibited a significantly lower ln(slope of recovery) than the 45‐min treatment (*p* = .002). Similarly, the mean damage assessed with *F*_V_/*F*_M_ 2 days after treatment was significantly greater in the 90‐min group compared to the 45‐min group (*p* = .006).

**Figure 3:**
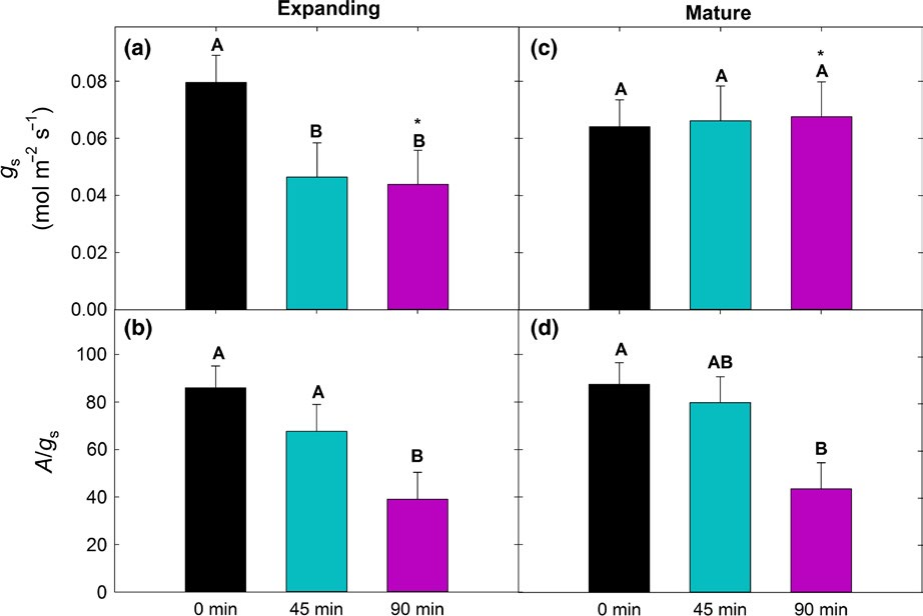
Mean post‐treatment stomatal conductance (*g*_s_) and intrinsic water‐use efficiency (*A*/*g*_s_) for 0‐min (controls), 45‐min, and 90‐min groups. Different uppercase letters represent significant differences among treatments within leaf age class. Error bars represent ± *SE*

Although mean post‐treatment stomatal conductance (*g*_s_) of expanding leaves exhibited a decline similar to that of photosynthesis (*A*, Figure 1), mean post‐treatment *g*_s_ of mature leaves did not significantly differ among treatments (*p* > .05, Figure 3a,c). This influenced mean post‐treatment intrinsic water‐use efficiency (*A*/*g*_*s*_) where expanding leaves of the 90‐min group had a significantly lower mean *A*/*g*_*s*_ than the control and 45‐min groups, and mature leaves of the 90‐min group had significantly lower mean *A*/*g*_*s*_ than the controls (Figure 3b,d).

**Figure 4:**
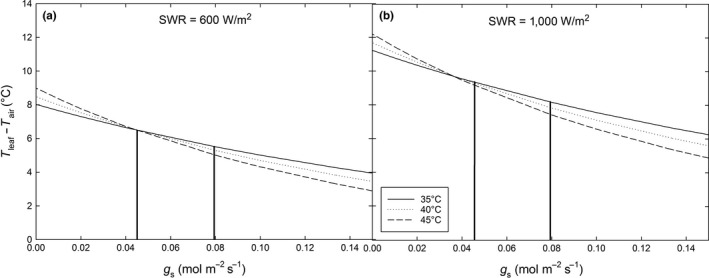
Modeled relationships between stomatal conductance (*g*_s_) and leaf temperature (*T*_leaf_) – air temperature (*T*_air_) at *T*_air_ = 35°C, 40°C, and 45°C at short‐wave radiation (SWR) of 600 W/m^2^ (a) and 1000 W/m^2^ (b) to represent partial and full sun conditions, respectively. Vertical lines indicate mean post‐treatment *g*_s_ values of controls (0.0796 mol·m^−2^·s^−1^) and of the 45‐min and 90‐min groups (0.0451 mol·m^−2^·s^−1^ for expanding leaves). Incident photosynthetically active radiation (PAR) for each SWR level at a wavelength of 550 nm (average wavelength for the 400–700 nm PAR range) was ~1380 and ~2300 μmol·m^−2^·s^−1^, respectively. PAR is assumed ~50% of SWR (Britton & Dodd, 1976)

The leaf energy balance model results showed that T_leaf_‐T_air_ increased with decreasing *g*_s_ in both partial (600 W/m^2^) and full (1,000 W/m^2^) sun conditions (Figure 4a,b). *T*_leaf_ was consistently greater in full sun (Figure 4b) than partial sun (Figure 4a). Even at higher values of *g*_s_ (e.g., 0.15 mol·m^−2^·s^−1^), *T*_leaf_ in full sun at *T*_air_ of 35–45°C (Figure 4b) was 42.0–51.2°C. Mean post‐treatment *g*_s_ of expanding leaves of control plants was 0.0796 mol·m^−2^·s^−1^ and treated plants was 0.0451 mol·m^−2^·s^−1^ (Figure 3b). The model estimated that this observed heat stress‐induced reduction in *g*_s_ (denoted by vertical lines in Figure 4a,b) would yield at *T*_air_ of 35°C, *T*_leaf_ of 41.6°C in partial sun and *T*_leaf_ of 44.5°C in full sun; at *T*_air_ of 40°C, *T*_leaf_ of 46.6°C in partial sun and *T*_leaf_ of 49.5°C in full sun; and at *T*_air_ of 45°C, *T*_leaf_ of 51.7°C in partial sun and *T*_leaf_ of 54.5°C in full sun (Figure 4a,b).

### 3.2 Nonstructural carbohydrates (NSCs)

**Figure 5:**
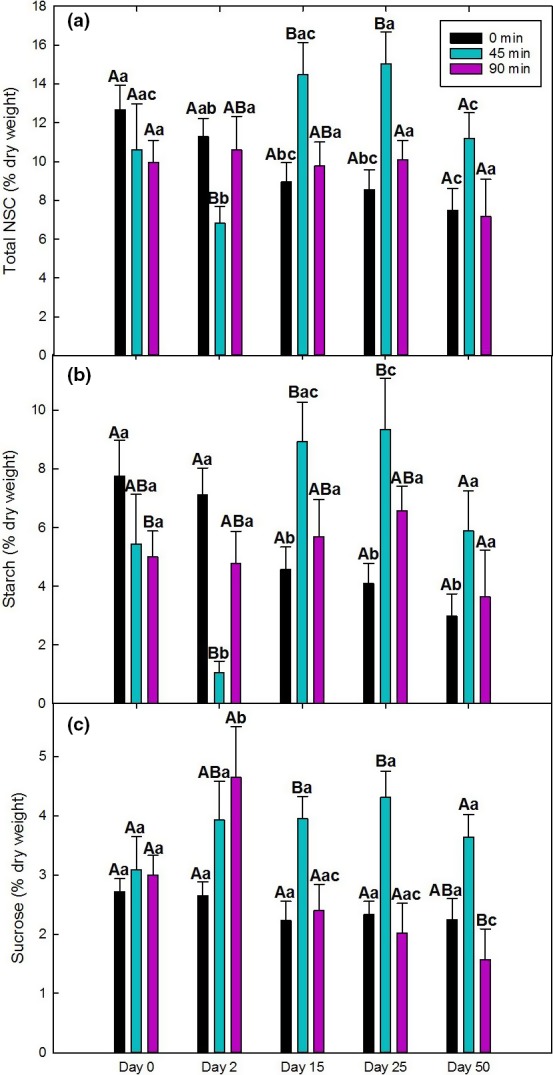
Time courses of mean total nonstructural carbohydrates (total NSC), starch, and sucrose of expanding and mature leaves from the same plants (*N* = 4) of the 0‐min (controls), 45‐min, and 90‐min groups at 0, 2, 15, 25, and 50 days after treatment. Different uppercase letters represent significant differences between treatments within each day. Different lowercase letters represent significant differences between days within each treatment. Error bars represent ± *SE*

**Figure 6:**
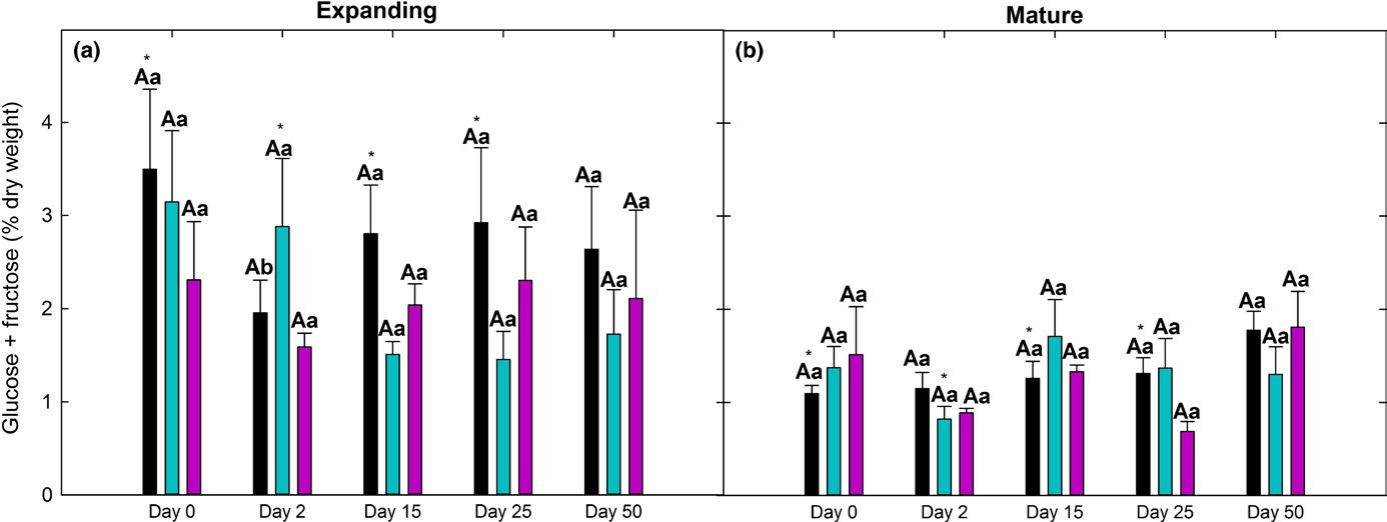
Time courses of glucose + fructose of expanding (a) and mature (b) leaves from the same plants (*N* = 4) of the 0‐min (controls), 45‐min, and 90‐min groups at 0, 2, 15, 25, and 50 days after treatment. Different uppercase letters represent significant differences among treatments within each day. Different lowercase letters represent significant differences among days within each treatment. Asterisks represent significant differences between leaf age classes. Error bars represent ± *SE*

NSC dynamics differed significantly across all three treatment groups (controls (0 min), 45, 90 min; Figures 5 and 6). Interactions among the main effects (age, treatment, day) on total NSC, starch, sucrose, and glucose + fructose are summarized in Table 2. Due to lack of significant leaf age‐related differences within day and treatment, total NSC, starch, and sucrose of expanding and mature leaves were combined by averaging over leaf age to simplify data visualization (Figure 5). Total NSC and starch in the controls steadily declined with time and were significantly less than day 0 values by day 50 (Figure 5a,b). In the 45‐min group, total NSC and starch significantly declined 2 days after treatment and were significantly less than that of any other day. At days 15 and 25, total NSC and starch of the 45‐min group significantly increased and became significantly greater than that of the control and 90‐min groups (Figure 5a,b). In contrast to the control and 45‐min groups, total NSC and starch of the 90‐min group did not significantly change with day (Figure 5a,b).

**Table 2:** Marginal F‐tests for total NSC, starch, sucrose, and glucose + fructose

	Total NSC				Starch				Sucrose				Glucose + Fructose			
	numDF	denDF	*F*‐value	*p*‐Value	numDF	denDF	*F*‐value	*p*‐Value	numDF	denDF	*F*‐value	*p*‐Value	numDF	denDF	*F*‐value	*p*‐Value
Intercept	1	79	850.78	**<.0001**	1	79	271.56	**<.0001**	1	79	620.693	**<.0001**	1	79	144.68	**<.0001**
Age	1	13	3.202	.0969	1	13	0.5649	.4657	1	13	4.833	**.0466**	1	13	52.792	**<.0001**
Treatment	2	13	4.280	**.0373**	2	13	0.123	.8856	2	13	11.66	**.0013**	2	13	1.974	.1784
Day	4	79	3.420	**.0124**	4	79	8.526	**<.0001**	4	79	4.385	**.0030**	4	79	2.481	.0505
Age × Treatment	2	13	0.442	.6523	2	13	1.015	.3899	2	13	0.66	.5333	2	13	0.511	.6113
Age × Day	4	79	1.846	.1282	4	79	1.276	.2867	4	79	1.010	.4072	4	79	2.497	**.0493**
Treatment × Day	8	79	5.682	**<.0001**	8	79	11.670	**<.0001**	8	79	3.222	**.0032**	8	79	0.702	.6893
Age × Treatment × Day	8	79	0.956	.4767	8	79	1.457	.1865	8	79	0.509	.8466	8	79	2.146	**.0408**

*N* = 4. Bolded *p*‐values indicate *p* < .05. NumDF and denDF are the degrees of freedom in the numerator and denominator, respectively.

Sucrose of the control and 45‐min groups did not significantly change with day (Figure 5c). In contrast, sucrose of the 90‐min group was significantly greater than that of controls 2 days after treatment before significantly declining and becoming significantly less than day 0 values at day 50 (Figure 5c). Although sucrose of the 45‐min group did not significantly change with day, it was significantly greater than that of controls and the 90‐min group at days 15 and 25 and that of the 90‐min group at day 50.

In contrast to total NSC, starch, and sucrose, glucose + fructose was significantly affected by leaf age (*F* = 52.792, *p* < .0001, Table 2) but did not significantly differ among treatments nor with day in either age class (Figure 6a,b). Glucose + fructose of controls was greater in expanding leaves than mature leaves at days 0, 15, 25, and in the 45‐min group at day 2. There were no age differences in the 90‐min group.

Pearson product–moment correlation indicated significant positive associations between photosynthesis and total NSC (*r* = .409, *p* = .00145, Table 3) and between photosynthesis and starch in the controls (*r* = .362, *p* = .00530, Table 3), and between photosynthesis and starch in the 45‐min group (*r* = .332, *p* = .0445, Table 3). In contrast, photosynthesis was not significantly related to any NSC component in the 90‐min group (*p* > .05, Table 3).

**Table 3:** Pearson correlation coefficients (*R*) and *p*‐values describing the relationship between mean expanding and mature leaf photosynthesis and NSC component (total NSC, starch, sucrose, glucose + fructose) within the 0‐min (controls), 45‐min, and 90‐min groups

		Total NSC	Starch	Sucrose	Glucose + Fructose
Photosynthesis					
0 min	*R*	**.409**	**.362**	.210	.141
	*p*‐Value	**.00145**	**.00530**	.113	.291
45 min	*R*	.318	**.332**	.0100	.161
	*p*‐Value	.0551	**.0445**	.953	.342
90 min	*R*	−.0427	−.0177	−.0920	.0606
	*p*‐Value	.805	.919	.594	.726

Bold values are significant *p *< .05.

## 4 Discussion

### 4.1 Implications for combined heat and drought scenarios

The sudden heat stress disrupted coordination between *A* and *g*_s_, leading to declining i*WUE* (*A*/*g*_s_) with increasing heat stress duration in both expanding and mature leaves (Figure 3). Under a combined heat and drought scenario expected throughout the 21st century (IPCC, 2014), sudden heat‐induced reductions in the ratio of carbon gain to water loss could accelerate the point at which drought, even in the absence of heat, would further restrict carbon gain (Chaves et al., 2002). Persistent reductions in *g*_s_ and therefore evaporative cooling (Ball, Cowan, & Farquhar, 1988; Monteith, 1981; Schymanski, Or, & Zwieniecki, 2013) of expanding leaves on heat‐stressed plants undergoing drought would place them at additional risk of further heat damage in closely spaced heat waves. The leaf energy balance model indicated that the extent to which *T*_leaf_ would increase in response to reduced *g*_s_ would be greatest at higher *T*_air_ (e.g., 45°C) compared to lower *T*_air_ (e.g., 35°C) and in full compared to partial sun (Figure 4a,b). *T*_leaf_ was especially sensitive to changes in *g*_s_ at relatively low *g*_s_, which would be expected during drought. For example, at *T*_air_ of 45°C and *g*_s_ of ~0.04 mol·m^−2^·s^−1^ as observed in expanding leaves of the treatment groups (Figure 3a), *T*_leaf_ would be ~51.7°C in partial sun (Figure 4a) and ~54.5°C in full sun (Figure 4b). These temperatures would cause significant damage, if not death, because the temperature causing a 90% reduction in initial *F*_V_/*F*_M_ estimated from chlorophyll fluorescence thermotolerance curves was 50.1°C in expanding leaves and 54.1°C in mature leaves (data not shown, see Marias et al., 2016). A factor not considered in the leaf energy balance model was stomatal sensitivity to VPD. Photosynthesis and *g*_s_ in *C. arabica* decline with increasing VPD (Oren et al., 1999) that typically ranges from 1.0 to 3.5 kPa throughout the day (Ronquim, Prado, Novaes, Fahl, & Ronquim, 2006). This VPD‐related decline in *g*_s_ would further increase *T*_leaf_ and therefore the risk of damaging heat stress. In contrast to expanding leaves, mature leaves maintained relatively high *g*_s_ and relatively high intercellular [CO_2_] across all treatments, which may have contributed to the faster recovery of *A* than *F*_V_/*F*_M_ in mature leaves (Figure 1). However, plants experiencing drought would not be able to maintain relatively high *g*_s_ without risking hydraulic failure and death (Adams et al., 2009). Therefore, recovery from heat stress would likely be inhibited during drought.

### 4.2 Greater sensitivity to heat stress in expanding leaves

Consistent with our first hypothesis, mature leaves showed faster recovery of photosynthesis than expanding leaves (Figure 1). However, the extent of damage as measured with photosynthesis at day 2 did not differ between leaf age classes nor treatment, suggesting that leaf age may influence the capacity to recover more than the extent of damage incurred, although both characteristics influence the ability to withstand heat stress (Escandón et al., 2016). Consistent with our second hypothesis, the 90‐min duration of heat stress resulted in both greater physiological damage and slower recovery than the 45‐min group as measured with *F*_V_/*F*_M_ and slower recovery as measured with photosynthesis (Figure 1). This was due to the apparent slower rate of *F*_V_/*F*_M_ recovery and greater damage at day 2 induced by the 90‐min treatment than the 45‐min treatment (Figures 1 and 2). Taken together, these results showed that leaf age affected the recovery rate of photosynthesis but not that of *F*_V_/*F*_M_, suggesting that PSII photochemistry may be conserved across different leaf ages in low light (Ishida, Uemura, Koike, Matsumoto, & Hoe, 1999) and that longer‐lived foliage may be protected by the greater capacity of older leaves to photosynthetically recover (Yamada et al., 1996). The declines in *F*_V_/*F*_M_ indicate that *F*_O_ increased and/or *F*_V_ decreased, both of which indicate stress‐induced changes to photochemistry including PSII inactivation, photodamage, heat dissipation, photoinhibition or damage to the oxygen evolving complex and water splitting system that disrupts electron donation to PSII reaction centers (Demmig, Winter, Krüger, & Czygan, 1987; Havaux, 1993; Maxwell & Johnson, 2000; Schreiber & Berry, 1977; Weis & Berry, 1987; Yamada et al., 1996; Yamashita & Butler, 1968).

In addition to their slower recovery, *g*_s_ of expanding leaves declined in the 45‐ and 90‐min treatments while that of mature leaves did not change with treatment. Besides reducing water loss, this response may reflect the impact of damaged membrane integrity on the ability to maintain *g*_s_ (Bita & Gerats, 2013). As discussed above, reduced *g*_s_ would exacerbate the effects of heat stress on expanding leaves by further increasing their temperature and putting them at greater risk of additional heat‐induced damage.

Interestingly, total NSC, starch, and sucrose content were not significantly different between leaf age classes (Figure 5) suggesting that these components were highly regulated. In contrast, glucose + fructose content was significantly greater in expanding leaves than mature leaves (Figure 6), which may occur because free sugars are needed as building blocks for structural carbohydrates (e.g., cellulose, lignin) in cell walls of expanding leaves, whereas sugars in mature leaves have already been allocated to structural carbohydrates required for structural support (Cavatte et al., 2012).

### 4.3 Impact of heat‐induced inhibition of flowering on NSC dynamics

In contrast to controls, none of the heat‐treated plants produced flowers or fruits. This is not surprising because coffee reproduction is highly sensitive to heat (Bita & Gerats, 2013; Camargo, 1985; Camargo, 2010). Our results showed that even a short, sudden heat stress event can inhibit reproduction and have negative consequences for *C. arabica* productivity (Bunn et al., 2014; Davis et al., 2012). The treatment temperature of 49°C for 45‐ and 90‐min durations may occur in the field because leaf temperatures can exceed air temperature by 15–20°C in sun grown *C. arabica* (Alvim & Kozlowski, 2013; Butler, 1977). This was supported by the leaf energy balance model (Figure 4). It is possible that *C. arabica* may acclimate and respond differently to gradual increases in average air temperature as opposed to a sudden heat stress event simulated in this study (Bauweraerts et al., 2013, 2014; Stone & Nicolas, 1995; Zou, 2009). However, dramatic aseasonal fluctuations in temperature are expected to become more frequent in the future (Filewod & Thomas, 2014). Further, elevated ambient [CO_2_] may partially mitigate the impacts of high temperature stress on *C. arabica* (Martins et al., 2016; Rodrigues et al., 2016) and *C. arabica* may be able to maintain adequate mineral balance under combined high temperature and elevated [CO2] situations (Martins et al., 2014).

Because NSCs are utilized for reproduction (e.g., flowers, fruits) in *C. arabica* (Cannell, 1976), the decline in total NSC and starch of controls (Figure 5a,b) was likely due to the subsequent formation of flowers and fruits (Chaves et al., 2012). Costa, Zambolim, and Rodrigues (2006) also observed reduced starch in high fruit‐bearing plants and no decrease in soluble sugars, consistent with the patterns of starch, sucrose, and glucose + fructose in the flower‐bearing control plants in this study.

Since fruit production is the strongest carbohydrate sink in *C. arabica* (Cannell, 1976) and competes with vegetative growth (Amaral, Matta, & Rena, 2001; DaMatta et al., 2007, 2008; Vaast et al., 2005), the lack of fruiting in the heat treatments should theoretically result in more NSC available for growth, storage, metabolic maintenance, and repair (Chapin, Schulze, & Mooney, 1990; Dietze et al., 2014; Kozlowski, 1992). This helps elucidate the patterns in total NSC and starch of the 45‐min group (Figure 5a,b). The sharp but transient reduction in total NSC and starch at day 2 coincided with the treatment‐induced reductions in photosynthesis and *F*_V_/*F*_M_, indicating significant heat stress‐induced damage and reductions in carbohydrate production (Wahid et al., 2007). The decline in starch with no significant shifts in sucrose and glucose + fructose at day 2 may indicate the allocation of NSC to metabolic maintenance and repair in response to the 45‐min treatment (Bita & Gerats, 2013). This may also be due to increased respiration (Way & Yamori, 2014), as well as the remobilization of starch from source leaves to roots (Blessing, Werner, Siegwolf, & Buchmann, 2015), although we did not measure NSC in roots. By days 15 and 25, total NSC, starch, and sucrose of the 45‐min group had significantly accumulated compared to controls, coinciding with the full recovery of photosynthesis and *F*_V_/*F*_M_. Due to the lack of demand for reproduction and repair by day 15 and later, carbon supply was greater than carbon demand (Génard et al., 2008) so starch was stored resulting in an increase in total NSC. By day 50, starch and total NSC levels of the 45‐min group declined to day 0 values suggesting that the stored NSC may have been utilized for renewed vegetative growth and metabolic maintenance.

Despite the greater damage induced by the 90‐min heat treatment compared to the 45‐min treatment (Figures 1 and 2), total NSC, starch, and glucose + fructose of the 90‐min group unexpectedly did not change significantly throughout the 50 days of the experiment (Figures 5 and 6). This may reflect a balance between NSC supply and sink demand in the 90‐min treatment. The supply of NSC was likely low due to the ongoing inhibition of photosynthesis and incomplete recovery of photochemistry (*F*_V_/*F*_M_) by day 50 (Figure 1). The sink demand in this treatment was also low due to the inhibition of reproduction. In contrast to the other NSC constituents that did not change with time in the 90‐min treatment, the transient increase in sucrose content at day 2 may reflect an initial repair or defense response to the substantial damage caused by the 90‐min treatment because sucrose has been linked to defense against ROS (Bita & Gerats, 2013), antioxidant production (Couée et al., 2006), osmotic adjustment (Cavatte et al., 2012), and stress response signaling (El Sayed, Rafudeen, & Golldack, 2014; Secchi & Zwieniecki, 2011, 2016; Sugio et al., 2009; Wang & Ruan, 2016). Also, soluble sugars such as sucrose are among the primary metabolites and osmolytes known to accumulate in response to heat stress (Wahid et al., 2007) and are necessary for protection from elevated temperature and maintaining water balance and membrane stability (Bita & Gerats, 2013; Farooq et al., 2008). Sucrose is translocated from source leaves to sink organs through the phloem, and its transient increase in plants subjected to 90 min of heat stress may also be associated with a disruption or inhibition of phloem transport (Blessing et al., 2015; Sala et al., 2012; Woodruff, 2014). The subsequent decline in sucrose after day 2 suggests that the demand for sucrose for repair, renewed growth, and ongoing metabolism was greater than the supply from photosynthesis, which was still inhibited on day 50.

### 4.4 Heat stress duration impacts on NSC dynamics

The observed effect of heat stress duration on PSII photochemistry and photosynthesis was associated with significant impacts on NSC dynamics. Although starch dynamics were presumably linked to reproduction in the control group, and a repair and storage response in the 45‐min group, the 90‐min group only exhibited shifts in sucrose content in response to treatment. This suggests that the greater duration of heat stress may have made starch inaccessible or overly energy intensive to utilize (Chapin et al., 1990; Dietze et al., 2014; Kozlowski, 1992) thereby resulting in the use of sucrose to allocate to repair. Escandón et al. (2016) also found that soluble sugars seemed more closely associated with plant responses to increasing number of days exposed to heat stress, although they did not measure starch. Interestingly, glucose + fructose was not significantly affected by day or treatment in the 45‐ and 90‐min groups (Figure 6), consistent with Lafta and Lorenzen (1995) that found no effect of temperature on sugar levels in potato and attributed this to coordinated control of sugar metabolism in response to high temperature stress.

Downregulation of photosynthesis has been associated with NSC accumulation in *C. arabica* (e.g., starch in DaMatta, Maestri, Mosquim, and Barros (1997), sucrose in Franck et al., 2006), while other studies have found no link between NSC and photosynthetic downregulation (Batista et al., 2011; DaMatta et al., 2016; Silva et al., 2004). In contrast, we found a positive relationship between starch and photosynthesis in controls and the 45‐min group (Table 3). These different results may be a consequence of previous studies of NSC and photosynthesis in *C. arabica* having examined NSC as a cause of photosynthetic regulation, whereas in this study, we examined changes in NSC dynamics as a consequence of heat stress‐induced inhibition of photosynthesis and other physiological processes. The lack of a significant relationship between photosynthesis and starch in the 90‐min group suggests a decoupling of starch dynamics and photosynthesis under greater heat stress duration, complicating predictions of plant carbon allocation under increasing temperature stress.

Currently, it is not well understood how photosynthate is partitioned under high temperature stress (Wahid et al., 2007). In this study, the differences in NSC dynamics among treatments emphasize that plant carbon utilization is influenced by heat stress duration and may be related to the capacity to recover (Filewod & Thomas, 2014; Teskey et al., 2015). The 45‐min treatment that induced less damage and faster recovery exhibited an NSC pattern of repair and storage as indicated by starch dynamics, while the 90‐min treatment that induced more damage and slower recovery exhibited an NSC pattern of repair and/or phloem transport inhibition as indicated by sucrose dynamics. Expanding leaves were more sensitive to heat stress, exhibited by slower photosynthetic recovery and lower stomatal conductance with increasing heat stress duration, a response likely to exacerbate heat stress effects during combined heat and drought scenarios. Reproduction and NSC dynamics are tightly linked in *C. arabica* and the heat‐induced inhibition of flowering significantly impacted NSC allocation dynamics, making the timing of heat stress at key developmental stages such as reproduction critical for interpreting and predicting responses to heat stress. The investigation of the impacts of heat stress duration and leaf age on NSC dynamics and recovery are essential for understanding plant carbohydrate metabolism and how *C. arabica* may respond to future climate change scenarios.

## Conflict of Interest

None declared.
