# Supplementary material for: Impacts of leaf age and heat stress duration on photosynthetic gas exchange and foliar nonstructural carbohydrates in Coffea arabica
Source: Ecol Evol. 2017 Jan 29;7(4):1297–310. doi: 10.1002/ece3.2681 (PMC5306013; doi:10.1002/ece3.2681)
Supplement: Supplementary file 1 [file ECE3-7-1297-s001.docx]

**SUPPORTING INFORMATION**

The modified leaf energy balance equation from Sridhar & Elliott (2002), Monteith & Unsworth (2007), and Jones (2013) to solve for T_leaf_ – T_air_ was:

$$T_{leaf}-T_{air}= \frac{\frac{{\gamma_{m}R}_{n}r_{bl}}{\rho C_{p}}-VPD}{s+ \gamma_{m}}$$

Symbols and definitions are in Table S2. The equation is based on the principle that for a leaf at steady state or equilibrium, the amount of energy entering the leaf via solar radiation and ambient heat is equal to the amount of energy that exits the leaf via heat loss, reflected light, and transpiration (e.g. Knoerr & Gay, 1965).

**Table S1.** Average daily daytime and nighttime air temperature, relative humidity, and maximum photosynthetically active radiation (PAR) of the greenhouse during the two experimental rounds in the summer of 2014.

|  | 21 July 2014 | 19 August 2014 |
| --- | --- | --- |
| Average daily daytime temperature  (°C) | 24.2 | 22.3 |
| Average daily nighttime temperature  (°C) | 18.3 | 18.0 |
| Average daily daytime relative humidity  (%) | 64.1 | 63.5 |
| Average daily nighttime relative humidity  (%) | 80.2 | 77.5 |
| Daily maximum PAR (µmol m^-2^ s^-1^) | 319.9 | 332.8 |

**Table S2.** Symbols, definitions, units, and equations of parameters used in the leaf energy balance model.

| Symbol | Definition | Units | Equation | Definition of symbols in equation |
| --- | --- | --- | --- | --- |
| T_leaf_ | leaf temperature | °C |  |  |
| T_air_ | air temperature | °C |  |  |
| $\gamma$_m_ | modified psychometric constant | kPa K^-1^ | $\gamma_{m}=\gamma( \frac{r_{st}}{r_{bl}} )$ | r_st_ = stomatal resistance (s m^-1^), r_bl_ = boundary layer resistance (s m^-1^) |
| R_n_ | net radiation | W m^-2^ | $R_{n}= {SWR}_{abs}+ {LWR}_{in}-{LWR}_{out}$ | SWR_abs_ = absorbed short wave radiation (W m^-2^), LWR_in_ = incoming long-wave radiation (W m^-2^), LWR_out_ = outgoing long-wave radiation |
| r_bl_ | leaf boundary layer resistance | s m^-1^ | $r_{bl}=\frac{1}{g (u \frac{j}{d(1-j)}}$ | j = 0.5 for a flat leaf, g = 0.00662 for a flat leaf, *u* = wind speed, d = leaf length in the direction of wind |
| VPD | water vapor pressure deficit of the air | kPa | $VPD = e_{sat}- e_{a}$ | e_sat_ = saturation vapor pressure $= a e^{\frac{b T_{air}}{T_{air}+z}}$, where a = 0.61121 kPa, b = 17.502, z = 240.97°C  ea = water vapor pressure of the air $=e_{sat}\frac{RH}{100}$, where RH = relative humidity (%) |
| ρ | density of dry air | kg m^-3^ | ρ = 1.292 kg m^-3^ |  |
| C_p_ | heat capacity of dry air | J kg^-1^ K^-1^ | C_p_ = 1010 J kg^-1^ K^-1^ |  |
| s | slope of the e_sat_/temperature curve | kPa | $s= \frac{e_{sat} b z}{{(T_{air}+z)}^{2}}$ | e_sat_ = saturation vapor pressure, b = 17.502, z = 240.97°C |


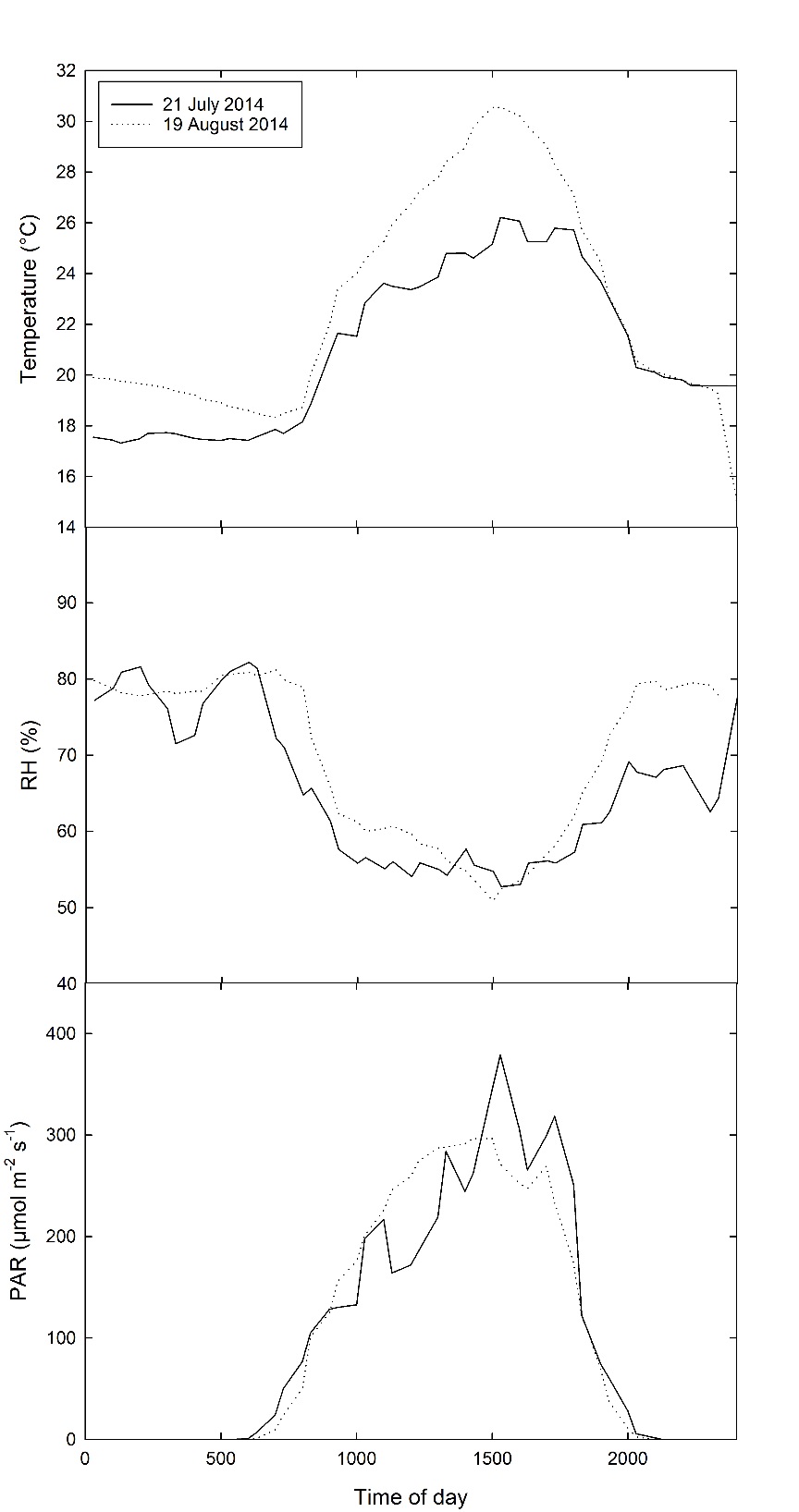


**Figure S1.** Air temperature, relative humidity (RH), and maximum photosynthetically active radiation (PAR) of the greenhouse during the two experimental rounds in the summer of 2014.


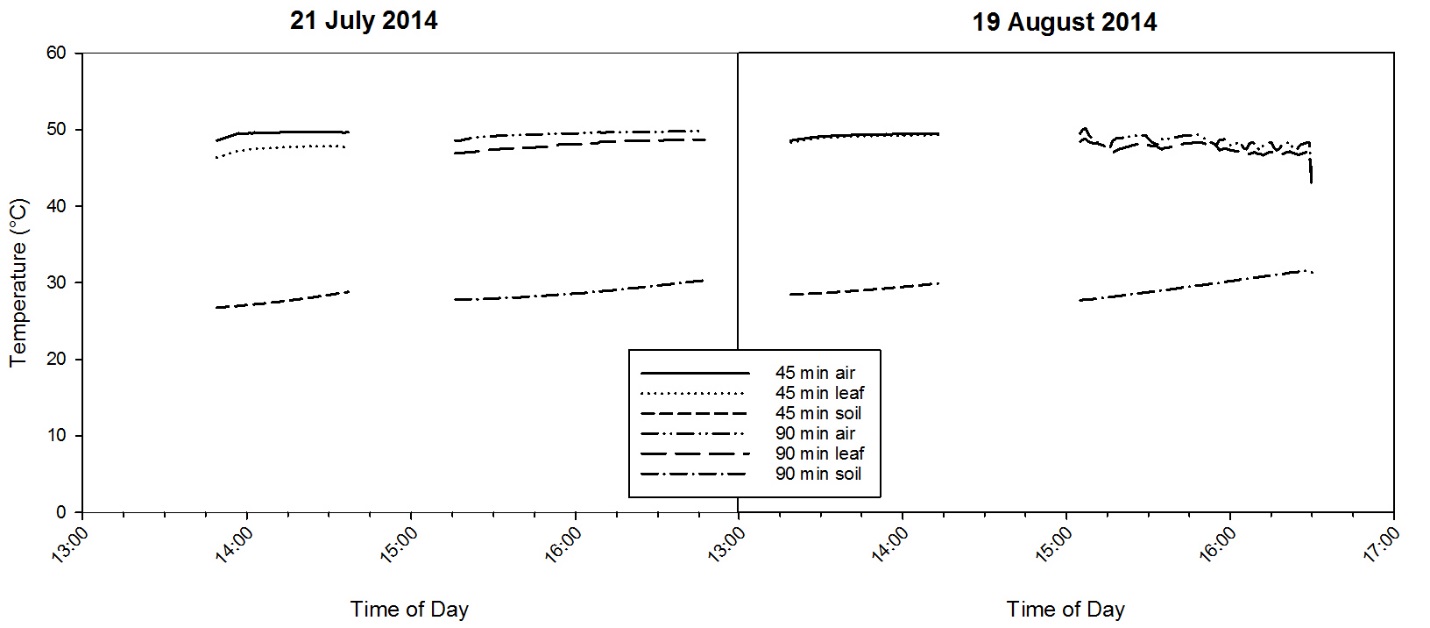


**Figure S2.** Air, leaf, and soil temperatures of plants during treatment exposure to 49°C for 45 min and 90 min in the growth chamber on 21 July 2014 and 19 August 2014.
